# A Chitin-binding Protein Purified from *Moringa oleifera* Seeds Presents Anticandidal Activity by Increasing Cell Membrane Permeability and Reactive Oxygen Species Production

**DOI:** 10.3389/fmicb.2017.00980

**Published:** 2017-06-06

**Authors:** João X.S. Neto, Mirella L. Pereira, Jose T. A. Oliveira, Lady C. B. Rocha-Bezerra, Tiago D. P. Lopes, Helen P. S. Costa, Daniele O. B. Sousa, Bruno A. M. Rocha, Thalles B. Grangeiro, José E. C. Freire, Ana Cristina O. Monteiro-Moreira, Marina D. P. Lobo, Raimunda S. N. Brilhante, Ilka M. Vasconcelos

**Affiliations:** ^1^Department of Biochemistry and Molecular Biology, Federal University of CearaFortaleza, Brazil; ^2^Department of Biology, Federal University of CearaFortaleza, Brazil; ^3^School of Pharmacy, University of FortalezaFortaleza, Brazil; ^4^Department of Pathology and Legal Medicine, Federal University of CearaFortaleza, Brazil

**Keywords:** prospection, moringa, plant protein, antifungal, candidiasis

## Abstract

*Candida* species are opportunistic pathogens that infect immunocompromised and/or immunosuppressed patients, particularly in hospital facilities, that besides representing a significant threat to health increase the risk of mortality. Apart from echinocandins and triazoles, which are well tolerated, most of the antifungal drugs used for candidiasis treatment can cause side effects and lead to the development of resistant strains. A promising alternative to the conventional treatments is the use of plant proteins. *M. oleifera* Lam. is a plant with valuable medicinal properties, including antimicrobial activity. This work aimed to purify a chitin-binding protein from *M. oleifera* seeds and to evaluate its antifungal properties against *Candida* species. The purified protein, named *Mo*-CBP_2_, represented about 0.2% of the total seed protein and appeared as a single band on native PAGE. By mass spectrometry, *Mo*-CBP_2_ presented 13,309 Da. However, by SDS-PAGE, *Mo*-CBP_2_ migrated as a single band with an apparent molecular mass of 23,400 Da. Tricine-SDS-PAGE of *Mo*-CBP_2_ under reduced conditions revealed two protein bands with apparent molecular masses of 7,900 and 4,600 Da. Altogether, these results suggest that *Mo*-CBP_2_ exists in different oligomeric forms. Moreover, *Mo*-CBP_2_ is a basic glycoprotein (pI 10.9) with 4.1% (m/m) sugar and it did not display hemagglutinating and hemolytic activities upon rabbit and human erythrocytes. A comparative analysis of the sequence of triptic peptides from *Mo*-CBP_2_ in solution, after LC-ESI-MS/MS, revealed similarity with other *M. oleifera* proteins, as the 2S albumin *Mo*-CBP_3_ and flocculating proteins, and 2S albumins from different species. *Mo*-CBP_2_ possesses *in vitro* antifungal activity against *Candida albicans*, *C. parapsilosis*, *C. krusei*, and *C. tropicalis*, with MIC_50_ and MIC_90_ values ranging between 9.45–37.90 and 155.84–260.29 μM, respectively. In addition, *Mo*-CBP_2_ (18.90 μM) increased the cell membrane permeabilization and reactive oxygen species production in *C. albicans* and promoted degradation of circular plasmid DNA (pUC18) from *Escherichia coli*. The data presented in this study highlight the potential use of *Mo*-CBP_2_ as an anticandidal agent, based on its ability to inhibit *Candida* spp. growth with apparently low toxicity on mammalian cells.

## Introduction

*Candida* species encompass a group of yeast that normally lives on the skin and mucous surfaces of healthy individuals (Mavor et al., 2005). Fortunately, just around 10% of approximately 200 *Candida* species identified to date are considered pathogenic to man. Most medically important *Candida* species include *C. albicans*, *C. glabrata*, *C. parapsilosis*, *C. tropicalis*, and *C. krusei*, which colonize the human body surface (Miceli et al., 2011). Candidiasis is the name given to the infections caused by the yeasts that belong to the genus *Candida* and avoidance of this disease constitutes a big challenge for modern medicine, with a significant impact on morbidity and overall mortality, especially among immunocompromised patients (Gil-Alonso et al., 2016). Incidentally, *C. albicans* is the principal causative agent of candidiasis (Pierce et al., 2015). The indiscriminate use of antibiotics, the incidence of diabetes mellitus type 1 and 2, and diseases or conditions which debilitate the immune system (chemotherapy, seropositive individuals, etc.) are within the factors associated with the increasing risk of developing candidiasis. In addition, patients subjected to surgery associated with the use of probes are also routinely affected by this infection (Schelenz, 2008; Achkar and Fries, 2010; Sanitá et al., 2014; Zhang et al., 2014; Alnuaimi et al., 2015).

Antifungal drugs currently available on the market for candidiasis treatment are often effective, but they can lead to the development of resistant strains, which could compromise the treatment efficiency. Drug resistance is related to three mechanisms: (1) decrease of intracellular drug concentration, (2) drug target alterations, and (3) metabolic bypasses (Sanglard, 2016). Furthermore, there are studies showing that these compounds may promote nephrotoxic (Niemirowicz et al., 2016), teratogenic (Dimopoulou et al., 2017), and cardiotoxic effects (Koch et al., 2015). Thus, it is very important to search for new compounds with antifungal activity, allowing the development of drugs and/or alternative treatments (Vandeputte et al., 2012; Salas et al., 2015). In this context, plants emerge as promising sources of bioactive compounds against *Candida* spp., since they have a large variety of primary (especially proteins) and secondary metabolites that are effective in defending plants against herbivores and/or pathogens (Wong et al., 2010). For instance, several plant proteins are known to possess harmful effects against *Candida* spp., which led to the suggestion of their biotechnological applications toward the candidiasis treatment. In this regard, chitin-binding proteins (CBP) can be highlighted due to their ability to interact with chitin, the main structural polysaccharide of fungal cell wall (Kanokwirron et al., 2008). The presence of chitin in fungal cell wall, where it performs relevant structural role for cell survival, and its absence in human cells, heighten chitin as a promising target for new antifungal drugs (Preechasuth et al., 2015; Campoy and Adrio, 2016). In literature, there are some reports on the antifungal effect of CBP against phytopathogenic fungi (Wang et al., 2012; Batista et al., 2014; Freitas C. D. et al., 2016), as well as against *Candida* spp. (Bertini et al., 2012; Gomes et al., 2012; Berthelot et al., 2016). Therefore, CBP are promising agents that have the potential to be used as antifungal compounds.

*Moringa oleifera* Lamarck (Moringaceae) is a fast-growing perennial species native to northeastern India that has a small to medium stature and is adapted to a variety of climates (Anwar et al., 2007). This species is well known in the world, particularly in tropical and subtropical regions, for its flocculation, nutritional, and pharmacological properties (Ndabigengesere et al., 1995; Santos et al., 2015; Freitas J. H. et al., 2016). Recently, our research group purified two CBP from *M. oleifera* seeds, eluted from a cation-exchange matrix with 0.5 M and 0.6 M NaCl, respectively, named *Mo*-CBP_3_ and *Mo*-CBP_4_ (*Mo*: *M. oleifera*; CBP: “Chitin-Binding Protein”), both of glycoprotein nature (Pereira et al., 2011; Gifoni et al., 2012). *Mo*-CBP_3_ displayed *in vitro* antifungal activity against *Fusarium solani*, *F. oxysporum*, *Colletotrichum musae*, and *C. gloesporioides*, phytopathogenic fungi to economically and nutritionally important crops (Batista et al., 2014; Freire et al., 2015), whereas *Mo*-CBP_4_ showed anti-inflammatory and antinociceptive activities in rats (Pereira et al., 2011). In our present investigation on *M. oleifera* seed proteins, we found an additional CBP eluted at a lower NaCl concentration (0.4 M) from the cation-exchange matrix, named *Mo*-CBP_2_, which displayed antifungal activity against *Candida* species. In this study, a purification protocol of *Mo*-CBP_2_ and its biochemical properties are presented. In addition, to evaluate the antifungal mode of action of *Mo*-CBP_2_, its ability to increase the cell membrane permeability and to induce endogenous production of reactive oxygen species in *C. albicans*, used as a model, were analyzed, as well as its DNAse activity. In view of the potential biotechnological applications of *Mo*-CBP_2_ as an antifungal agent, the hemolytic activity on red blood cells (cytotoxicity) was also investigated.

## Materials and Methods

### Biological Material and Chemical Reagents

*Moringa oleifera* seeds were collected from trees at the Campus do Pici of the Federal University of Ceará – UFC (Ceará, Brazil) under authorization (number: 47766) of the Chico Mendes Institute for Conservation of Biodiversity – ICMBio. A voucher specimen (N EAC34591) was deposited in the Prisco Bezerra Herbarium (UFC). The yeasts *C. albicans* (ATCC 10231), *C. parapsilosis* (ATCC 22019), *C. krusei* (ATCC 6258), and *C. tropicalis* (clinical isolate) were obtained from the culture collection of the Laboratory of Emergent and Reemergent Pathogens – LAPERE, Department of Pathology and Legal Medicine, UFC. Rabbit blood was obtained from animals of colonies maintained at the UFC. Human blood samples (ABO system) were obtained from healthy donors in the blood bank HEMOCE (Hemotherapy Center of Ceará). Experimental protocols were approved by the Ethics Committee of UFC, Brazil (protocol number: 77/2016).

Molecular mass markers, chromatographic matrices, IPG buffer, and immobilized pH gradient gel strips were obtained from GE Healthcare Life Sciences (New York, NY, United States). All other chemicals were purchased from Sigma-Aldrich Co. (St. Louis, MO, United States).

### Protein Quantification

Quantification of soluble protein was determined following the method described by Bradford (1976), using bovine serum albumin (BSA) as a protein standard. Absorbance at 280 nm was also used to detect the presence of protein in the chromatographic eluates.

### Purification of a Chitin-binding Protein from *M. oleifera* Seeds (*Mo*-CBP_2_)

Purification of *Mo*-CBP_2_ followed the procedure described by Gifoni et al. (2012), with modifications. Defatted seed flour was extracted with 0.05 M Tris-HCl buffer, pH 8.0, containing 0.15 M NaCl (1:10, m/v), for 3 h at 4°C under constant stirring. The resulting suspension was filtered and centrifuged at 15,000 × *g*, 4°C, 30 min. The supernatant was exhaustively dialyzed against distilled water at 4°C and centrifuged again under the same conditions. An aliquot of the supernatant (albumin fraction; 1.0 g) was loaded on a chitin column (3.5 cm × 20.0 cm) previously equilibrated with the above buffer. The *M. oleifera* chitin-binding proteins (*Mo*-CBPs) were eluted with 0.05 M acetic acid, pooled, dialyzed against distilled water at 4°C, and lyophilized. *Mo*-CBPs (400 mg) were dissolved in 20 mL of 0.05 M sodium acetate buffer, pH 5.2, and applied to a CM-Sepharose^TM^ Fast Flow column, pre-equilibrated with the same buffer. The adsorbed proteins were recovered by stepwise elution with increasing NaCl concentrations. *Mo*-CBP_2_ (*Mo*: *M. oleifera*; CBP: Chitin-Binding Protein) was dialyzed against distilled water at 4°C and subject to further analysis.

### Characterization of *Mo*-CBP_2_

#### Polyacrylamide Gel Electrophoresis Analysis

The purity of *Mo*-CBP_2_ was observed after polyacrylamide gel electrophoresis (PAGE) in the absence (native-PAGE) and presence of sodium dodecyl sulfate (SDS-PAGE). Native-PAGE was performed according to Reisfeld et al. (1962). *Mo*-CBP_2_ (5.0 μg) was loaded on 15% (m/v) polyacrylamide gel (10.0 cm × 8.0 cm) prepared in 1.5 M potassium acetate, pH 4.3. SDS-PAGE was performed as previously described (Laemmli, 1970). *Mo*-CBP_2_ samples (5.0 μg) were prepared in the sample buffer, in the presence or absence of 8% (v/v) 2-mercaptoetanol (2-ME) or 0.1 M dithiothreitol (DTT), and boiled at 98°C for 4 h. The samples were loaded on 15% (m/v) polyacrylamide gel prepared in 0.025 M Tris-HCl buffer, pH 8.9, containing 1% (m/v) SDS. The protein bands were detected by staining with 0.025% (m/v) Coomassie Brilliant Blue R-250 in methanol, acetic acid, and water (1.0:3.5:8.0, v/v/v). Molecular mass standards (GE Healthcare) were also loaded on the gel. The electrophoretic profile of reduced *Mo*-CBP_2_ was analyzed by tricine-SDS-PAGE (Schägger and von Jagow, 1987). The samples (10.0 μg) were incubated in 0.1 M DTT for 4 h at 98°C and alkylated with 0.2 M iodoacetamide (IAA) for 45 min at room temperature, followed by additional treatment with 4% (v/v) 2-ME for 10 min at 98°C. The samples were loaded and the electrophoretic run was carried out. Proteins were stained as above. Low molecular mass markers (Sigma–Aldrich) were also used.

#### Mass Spectrometric Analysis

Electrospray ionization mass spectrometry (ESI-MS) was performed using a Synapt G1 HDMS mass spectrometer (Waters) which was coupled to a nanoUPLC system. The intact mass of *Mo*-CBP_2_ (1 mg/mL in 0.1% [v/v] formic acid) was fractioned by reverse-phase chromatography using a gradient from 3 to 70% (v/v) acetonitrile with 0.1% (v/v) formic acid on HSS T3 C18 column (1.8 μm, 75 μm × 20 mm). Data were collected using MassLynx 4.1 (Waters). The acquired MS data were processed using a maximum-entropy technique (MaxEnt) to obtain a deconvoluted spectrum (Ferrige et al., 1991).

#### Isoelectric Point Determination

Isoelectric focusing (IEF) of *Mo*-CBP_2_ was performed according to Görg et al. (2000). *Mo*-CBP_2_ (150.0 μg) was solubilized in 250 μL of rehydration buffer, incubated with an immobilized pH gradient gel strip (11 cm, pH 6.0–11.0), and left standing for 12 h at room temperature. IEF was performed on a Multiphor II Electrophoresis system (GE Healthcare) using a stepwise protocol: 200 V/60 min, 500 V/90 min, 5,000 V/2.5 h, and 10,000 V/5.5 h. After IEF, the gel strip was brought in contact with the equilibration buffer containing 2.5% (m/v) DTT, for 15 min, followed by incubation with 7.5% (m/v) IAA, for 15 min, and submitted to SDS-PAGE (15%) on a vertical system (16.0 cm × 18.0 cm, Hoefer SE 600 Ruby, GE Healthcare). Molecular mass markers were run in the same gel. Proteins were stained with colloidal Coomassie (Candiano et al., 2004) and visualized using the Image Master TM 2D Platinum v.7.0 (GE Healthcare).

#### Carbohydrate Determination

The presence of covalently bound carbohydrate in the *Mo*-CBP_2_ structure was evaluated by periodic acid-Schiff staining (Zacharius et al., 1969). Briefly, *Mo*-CBP_2_ (15.0 μg) electrophoresis was performed as described above, the gel was fixed in 7.5% (v/v) acetic acid for 2 h and immersed in 0.2% (v/v) periodic acid for 45 min at 4°C, followed by staining in the periodic acid-Schiff reagent for 45 min at 4°C. Next, the gel was submerged in a 0.5% (m/v) potassium metabisulfite solution prepared in 0.05 M HCl. Fetuin (30.0 μg) was used as positive control. The neutral sugar content of *Mo*-CBP_2_ was estimated spectrophotometrically by the phenol-sulfuric acid method with reference to D-glucose (DuBois et al., 1956).

#### Amino Acid Sequencing

The N-terminal sequence analysis was done using an Automated Protein Sequencer (Shimadzu PPSQ-23A) via Edman degradation. The phenylthiohydantoin amino acid derivatives were detected at 269 nm after separation on an RP-HPLC C18 column (4.6 mm × 2.5 mm) under isocratic conditions, according to the supplier’s instructions. To obtain internal protein sequences, *Mo*-CBP_2_ (50.0 μg) was digested with 1.0 μg trypsin (Promega^^®^^) at 37°C for 16 h. The tryptic peptides were fractioned by reverse-phase chromatography using a gradient from 3 to 40% (v/v) acetonitrile with 0.1% (v/v) formic acid on HSS T3 C18 column (1.8 μm, 75 μm × 20 mm). After elution, data-dependent analysis (DDA) of peptides was performed using a Synapt HDMS mass spectrometer (nanoESI-Qq-*oa*TOF; Waters), where the three top peaks were subjected to MS/MS. The data were processed using ProteinLynx Global Server v.2.4 software (PLGS) and subjected to database search using the Mascot search engine (Perkins et al., 1999). MS/MS ion searches were performed against the NCBI non-redundant database (last accessed on January 14, 2017) using a significance threshold of *p* < 0.05. Searches for similar proteins in public sequence databases were performed using BLASTp (Altschul et al., 1990).

#### Evaluation of Hemagglutinating Activity

Hemagglutinating activity was assessed using untreated or trypsin-treated rabbit and human (ABO system) erythrocytes (Lis and Sharon, 1972). A serial 2-fold dilution (100 μL) of *Mo*-CBP_2_ (5.0 mg/mL in 0.15 M NaCl) in a 96-well plate was mixed with 100 μL of a 4% (v/v) erythrocyte suspension in 0.15 M NaCl from 1 h up to 24 h at 37°C (Raja et al., 2011). Hemagglutination was checked in comparison to a blank in which *Mo*-CBP_2_ had been omitted. The minimal protein concentration after serial dilution still promoting visible agglutination with naked eyes was taken to calculate the hemagglutinating activity.

### Evaluation of Antifungal Activity and Mode of Action of *Mo*-CBP_2_

#### *In Vitro* Antifungal Activity Test

The antifungal activity of *Mo*-CBP_2_ was tested against the yeasts *C. albicans*, *C. krusei*, *C. parapsilosis*, and *C. tropicalis*. The susceptibility of *Candida* spp. to *Mo*-CBP_2_ was evaluated using the broth microdilution method, as described by the Clinical and Laboratory Standards Institute (Clinical and Laboratory Standards Institute [CLSI], 2012), with modifications. Potato dextrose broth (PDB) medium and nystatin (EMS) were used instead of Roswell Park Memorial Institute (RPMI) medium and amphotericin B. Inocula (final concentration of 0.5 – 2.5 × 10^3^ CFU/mL) were prepared from 1-day-old cultures grown on potato dextrose agar (PDA) at 35°C in PDB medium. *Mo*-CBP_2_, *Mo*-CBP_3_ (Gifoni et al., 2012), *Mo*-CBP_4_ (Pereira et al., 2011) (0.1–1,346.46 μM), and nystatin (0.085–1,067.04 μM) were prepared in ultrapure water and sterilized using a 0.22 μm membrane filter. Aliquots (100 μL) of each yeast suspension (0.5–2.5 × 10^3^ CFU/mL) were incubated in 96-well flat plates with 50 μL of *Mo*-CBP_2_ and additional 50 μL of PDB medium (4-fold concentrated). Plates were incubated at 37°C for 24 h and the yeast growth was monitored at 620 nm using an automated microplate reader (Epoch, BioTek). Nystatin and 0.15 M NaCl were used as positive and negative controls, respectively. *Mo*-CBP_3_ and *Mo*-CBP_4_ were used as reference proteins, as they are also CBP from *M. oleifera* seeds. Yeast-free control was also included. The Minimum Inhibitory Concentrations (MIC_50_ and MIC_90_) were defined as the lowest protein or nystatin concentration capable of inhibiting 50% and 90% fungal growth, respectively.

#### Evaluation of Cell Membrane Integrity of *C. albicans* Cells after *Mo*-CBP_2_ Treatment

Plasma membrane permeabilization was measured by propidium iodide uptake (Regente et al., 2014). *C. albicans* cells (0.5–2.5 × 10^3^ CFU/mL) were cultured in the presence of 18.9 μM *Mo*-CBP_2_ or 11.11 μM nystatin (positive control), whose concentrations corresponding to their respective MIC_50_ calculated in this study, or 0.15 M NaCl (negative control), for 24 h at 37°C. The *Mo*-CBP_2_ and nystatin-treated cell suspensions (100 μL) were incubated with 0.001 M propidium iodide in 96-well microplates for 30 min at 30°C, under constant and moderate agitation. Next, the cells were observed under a fluorescence microscope (Olympus System BX 60; excitation wavelength, 400–500 nm; emission wavelength, 600–700 nm).

#### Evaluation of Reactive Oxygen Species (ROS) Production by *C. albicans* Cells after *Mo*-CBP_2_ Treatment

This was done according to Thordal-Christensen et al. (1997), with modifications (Batista et al., 2014). Aliquots (100 μL) of the cell suspensions (0.5–2.5 × 10^3^ CFU/mL) previously treated with *Mo*-CBP_2_, nystatin or NaCl, as described above, were incubated with 100 μL of 3,3′-diaminobenzidine (DAB, 1.0 mg/mL in H_2_O) for 1 h at 30°C. Next, the cells were observed under a light microscope (Olympus System Microscope BX 60).

#### Assessment of DNase Activity of *Mo*-CBP_2_

The effect of *Mo*-CBP_2_ on DNA degradation was assessed as previously described (Tomar et al., 2014a), using the pUC18 plasmid of *E. coli*. The plasmid (500.0 ng) was incubated with *Mo*-CBP_2_ (500.0 ng) in 0.05 M Tris-HCl buffer, pH 7.4, in a total reaction volume of 20 μL, for 1 h at 37°C. Recombinant DNase I (2 units, RNase-free, Roche), BSA (500.0 ng), and 0.05 M Tris-HCl buffer, pH 7.4, all equally incubated with the plasmid, were used as controls. The samples were loaded on 1% (m/v) agarose gel (10.0 cm × 10.0 cm), prepared in the TAE buffer (0.05 M Tris-HCl, 0.02 M sodium acetate, and 0.001 M EDTA), mounted on a horizontal system (MultiSUB Midi10, Cleaver Scientific), and run at 6 V/cm. The DNA bands were visualized under UV light after staining the gel with 0.5 μg/mL ethidium bromide solution for 30 min.

### Evaluation of Hemolytic Activity of *Mo*-CBP_2_

The hemolytic assay was performed using rabbit and human (ABO system) erythrocytes collected in heparinized tubes (Choi and Lee, 2014). Erythrocytes were separated from plasma by centrifugation (3,000 × *g*, 10 min, 25°C) and washed three times with 0.15 M NaCl. An aliquot (100 μL) of a 4% (v/v) suspension was incubated with an equal volume of *Mo*-CBP_2_ (1.9–1000 μg/mL), both prepared in 0.15 M NaCl, for 1 h at 37°C. After incubation, the mixtures were centrifuged at 3,000 × *g* for 10 min at 25°C and aliquots of the supernatants transferred to Eppendorf tubes. Absorbance readings were taken at 414 nm (spectrophotometer Novaspec II, Pharmacia) to monitor the release of hemoglobin. Triton X-100 (0.1%, v/v) and 0.15 M NaCl were used as positive and negative controls, respectively. The hemolysis index was calculated using the following equation: Hemolysis (%) = ([A_protein_ – A_NaCl_]/[A_Triton_ – A_NaCl_]) × 100, where A means absorbance at 414 nm.

### Statistical Analysis

Data were obtained from three independent experiments, each one done in triplicate. The results are expressed as the mean ± standard deviation (SD). Tukey’s test was used to compare means and the results were considered to be significant at *p* < 0.05. GraphPad Prism 5.02 software was used for statistical analysis.

## Results

### Purification of *Mo*-CBP_2_

*Mo*-CBP_2_ was purified by a combination of albumin separation from the crude extract and two chromatographic steps. The crude extract obtained from *M. oleifera* seeds presented 205.41 mg protein/g of defatted seed. Exhaustive dialysis of the crude extract against distilled water produced the albumin fraction that concentrated approximately 55% of the soluble proteins. Chromatography of albumin on a chitin column produced a chitin-binding protein peak (*Mo*-CBPs) (Supplementary Figure 1A), which represented 23.2% of the seed extract soluble proteins. *Mo*-CBPs chromatographed on a CM-Sepharose^TM^ Fast Flow column emerged as a through fraction and three adsorbed protein peaks (Supplementary Figure 1B). *Mo*-CBP_2_ was recovered after elution of the adsorbed proteins from the column with 0.4 M NaCl included in 0.05 M sodium acetate buffer, pH 5.2, yielding 0.32 mg protein/g of the defatted flour, or 0.2% of the soluble proteins of the seed crude extract (**Table 1**).

**Table 1:** Purification of *Mo*-CBP_2_.

Purification steps^a^	Total protein (mg)^b^	Yield (%)^c^
Crude extract	205.41 ± 2.55	100
Albumin	112.45 ± 3.00	54.7
Chitin chromatography	47.57 ± 5.46	23.2
CM-Sepharose chromatography (*Mo*-CBP_2_)	0.32 ± 0.02	0.2

*Results are the mean ± standard deviation of six similar runs. ^a^Purification steps are described in Materials and Methods. ^b^Total amount of protein recovered from 1 g of defatted flour from *M. oleifera* seeds. ^c^Protein recovery at each purification step (crude extract, 100%)*.

### Molecular and Physicochemical Properties of *Mo*-CBP_2_

#### Purity, Molecular Mass, Isoelectric Point, and Carbohydrate Content

A single polypeptide band appeared after native electrophoresis of *Mo*-CBP_2_ (Supplementary Figure 2, inset), suggesting that the purification procedure employed yielded a contaminant-free, homogenous protein. ESI-MS of the intact protein revealed a major peak at 13,309 Da (Supplementary Figure 2). However, SDS-PAGE of *Mo*-CBP_2_ under non-reducing conditions showed a 23,400 Da protein band (Supplementary Figure 3, lane 1). *Mo*-CBP_2_ treated with 8% (v/v) 2-ME or 0.1 M DTT presented the same prominent band of 23,400 Da and additional faint bands (Supplementary Figure 3, lanes 4 and 7). In tricine-SDS-PAGE, *Mo*-CBP_2_ treated with 0.1 M DTT, alkylated with 0.2 M IAA followed by treatment with 4% (v/v) 2-ME revealed the generation of two protein bands with apparent molecular masses of 7,900 and 4,600 Da (Supplementary Figure 4, lanes 2, 4, and 6). Two-dimensional electrophoresis analysis of *Mo*-CBP_2_ revealed the presence of a protein spot of 22,300 Da apparent molecular mass and isoelectric point (pI) of 10.9 (data not shown). Moreover, *Mo*-CBP_2_ was stained by periodic acid-Schiff’s reagent on SDS-PAGE, suggesting it is a glycoprotein (Supplementary Figure 5), confirmed by quantitative analysis that showed 4.1% (m/m) covalently linked neutral carbohydrates to the *Mo*-CBP_2_ structure.

#### Amino Acid Sequence

Edman degradation of *Mo*-CBP_2_ did not give any sequence, suggesting that its N-terminal residue was blocked. However, after tryptic hydrolysis of *Mo*-CBP_2_ followed by LC-ESI-MS/MS analysis of the resulting fragments, six peptides were identified (**Table 2**) and their sequences deposited in the Swiss-Prot database (access number: C0HKC5). By aligning the sequences of these fragments against the non-redundant protein sequence database of NCBI, limited to Brassicales for the first three peptides and Viridiplantae for the last three peptides, these *Mo*-CBP_2_ sequences were more closely related (75–100% identity) to other proteins purified from *M. oleifera*, as 2S albumin precursors (*Mo*-CBP_3_ isoforms: AHG99684.1; AHG99683.1; AHG99682.1), a flocculating protein (prf| | 2111235A), and MO 2.1 (AAB34890.1). Similarities (63–83%) were also found with 2S albumins of other plant species (*Capparis masaikai* [BAA12204.1, P80351.1], *Bertholletia excelsa* [ACI70207.1], *Ziziphus jujuba* [XP_015899022.1]).

**Table 2:** Amino acid sequences of tryptic peptides from *Mo*-CBP_2_ identified by LC-ESI-MS/MS.

Peptide sequence	Mass (Da)		Proteins (species)^a^	NCBI accession number	Identity (%)
	Experimental	Calculated			
			2S albumin precursor (*Moringa oleifera*)	AHG99684.1	100
CPSLR	863.3508	863.3743	MO 2.1 (*Moringa oleifera*)	AAB34890.1	100
			Flocculating protein (*Moringa oleifera*)	prf| | 2111235A	100
			MO 2.1 (*Moringa oleifera*)	AAB34890.1	100
QPDFQR	789.3526	789.3730	Flocculating protein (*Moringa oleifera*)	100
			2S albumin precursor (*Moringa oleifera*)	AHG99683.1	83
			2S albumin precursor (*Moringa oleifera*)	AHG99684.1	100
CCQQLR	1229.5258	1229.5200	2.1 protein (*Moringa oleifera*)	CAC69951.1	100
			Mabinlin (*Capparis masaikai*)	BAA12204.1	83
			2S albumin precursor (*Moringa oleifera*)	AHG99683.1	100
QQFQTHQR	1071.4878	1071.5210	2S albumin precursor (*Moringa oleifera*)	AHG99684.1	88
			2S albumin precursor (*Moringa oleifera*)	AHG99682.1	75
			2S albumin precursor (*Moringa oleifera*)	AHG99684.1	90
IPAICNLQPMR	1311.5858	1311.6790	2S albumin precursor (*Bertholletia excelsa*)	ACI70207.1	73
			Flocculating protein (*Moringa oleifera*)	prf| | 2111235A	100
			2S albumin precursor (*Moringa oleifera*)	AHG99684.1	95
QAVQLTHQQQGQVGPQQVR	2129.0552	2129.1089	Mabinlin-1 (*Capparis masaikai*)	P80351.1	63
			2S seed storage protein-like (*Ziziphus jujuba*)	XP_015899022.1	63

^a^*Results after the non-redundant protein sequence database (NCBI) searches. For the three first peptides from *Mo*-CBP_*2*_, the searches were restricted to Brassicales order. For the three last peptides, the searches were done within Viridiplantae*.

### Assessment of Hemagglutination Activity of *Mo*-CBP_2_

*Mo*-CBP_2_ did not agglutinate rabbit and human erythrocytes, even at 5.0 mg/mL. These results did not differ regardless of whether the erythrocytes were trypsin-treated or untreated.

### Antifungal Activity and Mode of Action of *Mo*-CBP_2_

*Mo*-CBP_2_ inhibited the development of *C. krusei*, *C. albicans*, *C. tropicalis*, and *C. parapsilosis* with MIC_50_ values varying from 9.45 to 37.90 μM, whereas *Mo*-CBP_3_ and *Mo*-CBP_4_, also CBP, had MIC_50_ between 261.67 and 310.06 μM. To cause 90% fungal growth inhibition, the MIC_90_ for *Mo*-CBP_2_, *Mo*-CBP_3_, and *Mo*-CBP_4_ were in the ranges of 155.84–260.29 μM, 560.32–600.23 μM, and 564.89–598.10 μM, respectively. MIC_50_ for nystatin varied from 11.11 to 22.23 μM and MIC_90_ from 55.55 to 133.38 μM (**Table 3**).

**Table 3:** Antifungal activity^a^ of chitin-binding proteins from *M. oleifera* seeds and nystatin against *Candida* species.

Fungal strains	*Mo*-CBP_2_	*Mo*-CBP_3_	*Mo*-CBP_4_	Nystatin
*C. albicans* ATCC 10231 MIC_50_ (μM)^b^ MIC_90_ (μM)^c^	18.90^A^ 169.50^A^	299.30^B^ 600.23^B^	290.27^B^ 598.10^B^	11.11^C^ 55.55^C^
*C. krusei* ATCC 6258 MIC_50_ (μM) MIC_90_ (μM)	9.45^A^ 155.84^A^	270.60^B^ 560.32^B^	261.67^B^ 564.89^B^	11.11^C^ 55.55^C^
*C. parapsilosis* ATCC 22019 MIC_50_ (μM) MIC_90_ (μM)	37.90^A^ 260.29^A^	310.06^B^ 570.65^B^	309.65^B^ 573.16^B^	22.23^C^ 133.38^C^
*C. tropicalis*^d^ MIC_50_ (μM) MIC_90_ (μM)	18.90^A^ 180.98^A^	303.98^B^ 588.26^B^	300.12^B^ 585.45^B^	22.23^C^ 133.38^C^

^a^*Antifungal activity was evaluated after 24 h incubation at 37°C. ^b,c^Minimum concentrations that inhibited 50 and 90% of fungal growth, respectively. ^d^Clinically isolated strain. Different capital letters in the row mean significant differences (*p* < 0.05, Tukey’s test) between data*.

**FIGURE 1:**
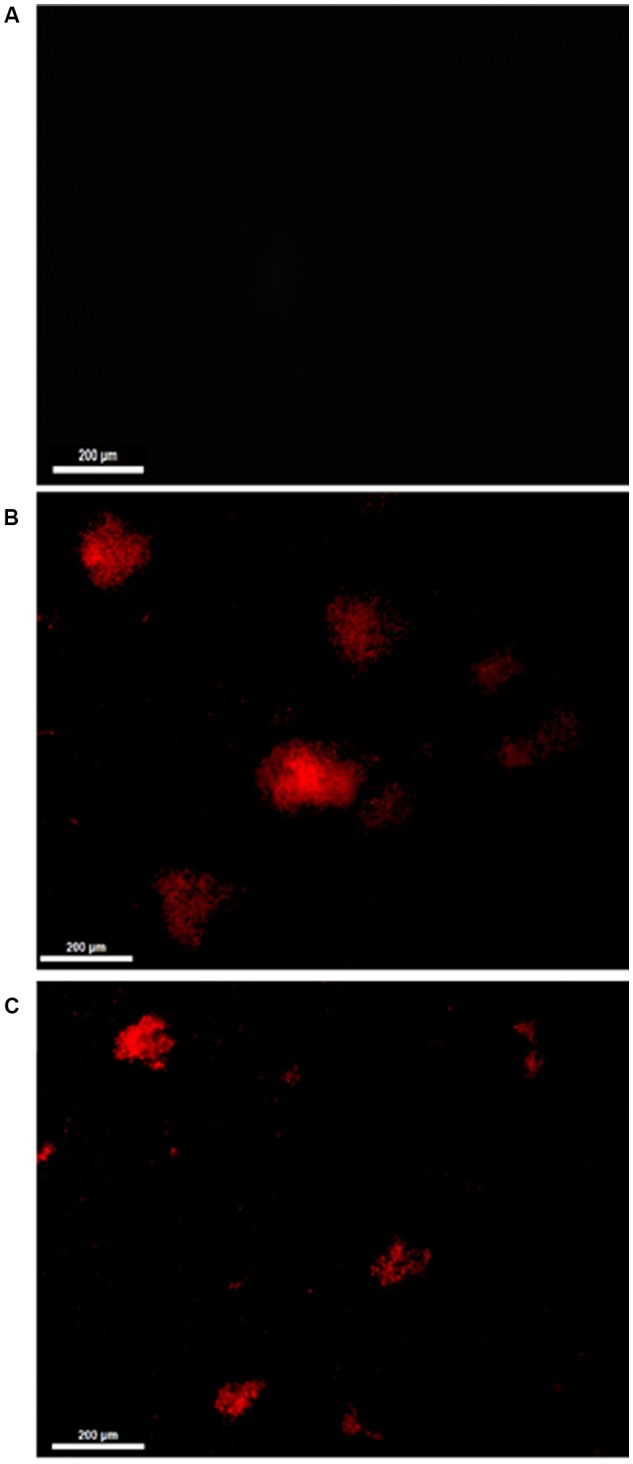
Fluorescence microscopy of *C. albicans* cells treated with 0.15 M NaCl **(A)**, 11.11 μM nystatin **(B)**, or 18.90 μM *Mo*-CBP_2_ **(C)**, followed by incubation with 0.001 M propidium iodide. Bars = 200 μm.

Treatment of *C. albicans*, used as a representative model, with 11.11 μM nystatin or 18.90 μM *Mo*-CBP_2_ for 24 h altered the cell membrane permeability as revealed by propidium iodide uptake (**Figure 1**).

**FIGURE 2:**
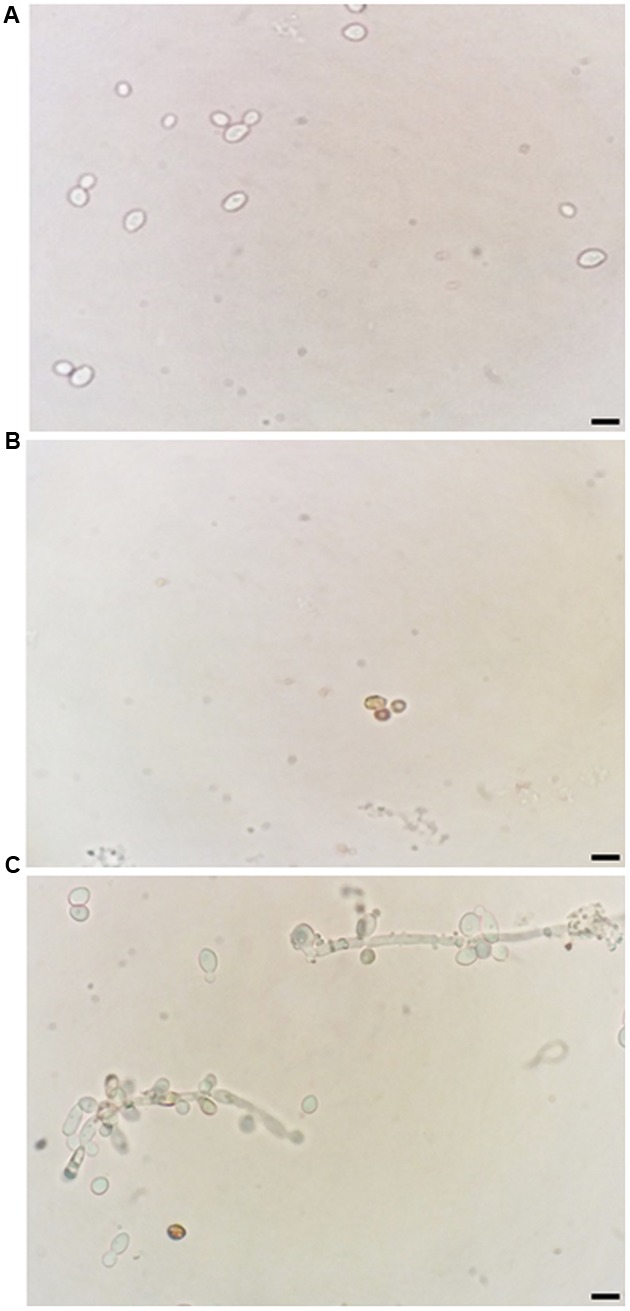
Induction of ROS generation in *C. albicans.* Light micrography of cells previously treated with 0.15 M NaCl **(A)**, 11.11 μM nystatin **(B)**, or 18.90 μM *Mo*-CBP_2_ for 24 h at 37°C **(C)**, followed by incubation with DAB. The presence of ROS was observed by dark staining (reddish-brown) reaction inside cells. Bars = 10 μm.

**FIGURE 3:**
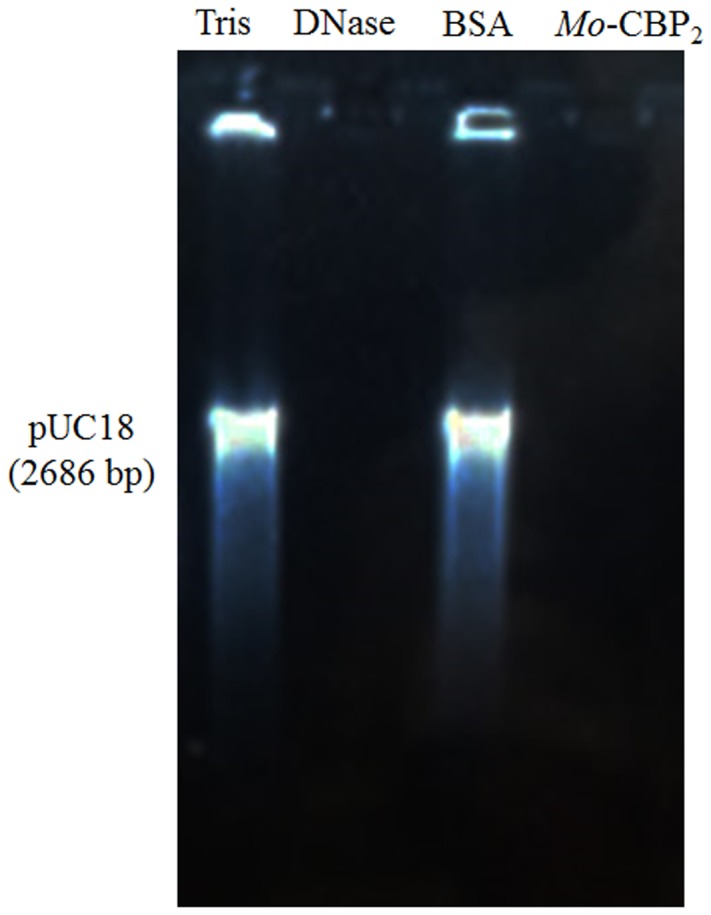
DNase activity of *Mo*-CBP_2._ The pUC18 plasmid (500.0 ng) of *E. coli* was incubated with *Mo*-CBP_2_ (500.0 ng); BSA (500.0 ng) and 0.05 M Tris-HCl buffer, pH 7.4 (negative controls); and the recombinant DNase I (2 units, positive control) for 1 h and loaded in 1% (m/mL) agarose gel electrophoresis. Gel was stained with ethidium bromide and observed under UV light.

In addition, ROS overproduction was observed, recognized as internal dark staining, after incubation of *C. albicans* cells with 11.11 μM nystatin or 18.90 μM *Mo*-CBP_2_ (**Figure 2**). Similar to the recombinant DNase I, *Mo*-CBP_2_ (500.0 ng) also promoted DNA degradation of the *E. coli* plasmid (pUC18), whereas both BSA and 0.05 M Tris-HCl buffer, pH 7.4, were inactive (**Figure 3**).

### Assessment of the Cytotoxicity Effect of *Mo*-CBP_2_

Bovine serum albumin and *Mo*-CBP_2_ did not exhibited any hemolytic activity on rabbit and human erythrocytes in all concentrations analyzed, contrary to the positive control, Triton X-100, which caused strong hemolysis on the tested erythrocytes (data not shown).

## Discussion

The World Health Organization (WHO) has long been concerned with the rational use of antibiotics and the emergence of super-resistant microorganisms. World Health Organization [WHO] (2015) launched the first “World Antibiotics Awareness Week” with the theme “Antibiotics: Handle with care,” from which a list with recommendations to prevent antibiotic resistance was produced. In consonance with this concern, several research groups have been searching for new antimicrobial agents to efficiently overcome the development of microbial resistance, devoid of side effects, and also not expensive. It has long been known that plants are rich sources of new compounds with potential for the treatment of various diseases, including infectious diseases. Studies exploring the mechanism of action and structure-activity aspects of such natural compounds are important toward the discovery and development of novel antimicrobial agents (Hayashi et al., 2013). In connection with this global antimicrobial resistance dilemma the current study reported on the purification and characterization of *Mo*-CBP_2_, and evaluated its anticandidal effect.

*Mo*-CBP_2_ analyzed by PAGE under native condition appeared as a single band protein. After tricine-SDS-PAGE under reducing conditions, *Mo*-CBP_2_ dissociated in two protein bands with apparent molecular masses of 7,900 and 4,600 Da, values that summed up (12,500 Da) result in a molecular mass near to that determined by mass spectrometry analysis (13,309 Da). However, *Mo*-CBP_2_ analyzed by SDS-PAGE under non-reducing conditions migrated as a 23,400 Da protein. These findings suggest the formation of dimer species of *Mo*-CBP_2_, with each monomer composed of the 7,900 and 4,600 Da subunits probably linked by dissulfide bond. The other CBP previously purified by our research group, *Mo*-CBP_3_ (Gifoni et al., 2012) and *Mo*-CBP_4_ (Pereira et al., 2011), also behaved as oligomeric proteins. Actually, several other similarities between *Mo*-CBP_2_ and *Mo*-CBP_3_ and *Mo*-CBP_4_ exist, like the molecular mass of the intact proteins (∼13,300, 12,200, and 11,800 Da, respectively) and subunits (∼4,600/7,900, 4,100/8,100, and 3,900/8,400 Da, respectively) (Pereira, 2014; Freire et al., 2015), which suggest that they are part of the same family of CBP. Similar oligomeric behavior was also observed for other proteins from *M. oleifera* seeds. Indeed, both the hemagglutinin MoL and the flocculating lectin cMoL presented molecular profiles similar to *Mo*-CBP_2_, appearing in dimer or trimer conformations (Katre et al., 2008; Luz et al., 2013). The *in silico* analysis of the recombinant protein MO 2.1 showed that the dimer form was the most stable structural arrangement (Pavankumar et al., 2014). Thus, it is plausible to suggest that the *M. oleifera* proteins aggregate into oligomers to attain a stable and lower energy structural state.

*Mo*-CBP_2_ is a glycoprotein as are other CBP from *M. oleifera* purified by our research group (Pereira et al., 2011; Gifoni et al., 2012). However, a difference in relation to *Mo*-CBP_3_ and *Mo*-CBP_4_ is that the carbohydrate content was approximately two times higher in *Mo*-CBP_2_. Another similar characteristic to *Mo*-CBP_3_ and *Mo*-CBP_4_ is that *Mo*-CBP_2_ does not present any hemagglutinating activity upon human or rabbit erythrocytes, contrary to Mol and cMol proteins (Katre et al., 2008; Luz et al., 2013). Therefore, *Mo*-CBP_2_ is a merolectin as it apparently possesses a unique carbohydrate binding site (Peumans and Van Damme, 1995) which binds to *N*-acetyl-D-glucosamine and its derived polysaccharide chitin, thus incapable of establishing bridges between two of more blood cells toward agglutinating them (Gifoni et al., 2012)

The MS/MS analysis done with *Mo*-CBP_2_ after tryptic digestion, as an attempt to disclose its primary structure, allowed identification of 6 peptide fragments that together comprise 55 amino acid residues, or approximately 45% of the total amino acid residues of the studied protein. Four cysteine (C) residues were present, which might form inter or intrachain disulphide bonds. Glutamine (Q) was the most abundant amino acid residue (about 31%), which could be related to dimer formation tendency of *Mo*-CBP_2_. It was previously reported that glutamine-rich regions may cause aggregation by formation of β-pleated sheets held together by hydrogen bonds (Perutz et al., 1994; Michelitsch and Weissman, 2000; Broin et al., 2002). In addition, the presence of glutamine at the N-terminus of *Mo*-CBP_2_ could also explain the failure to identify the protein sequence by Automated Edman degradation. Indeed, cyclization of N-terminal glutamine to pyroglutamate leads to a blocked chain, and this event has been described for several 2S albumins (Moreno et al., 2005). Particularly noteworthy is that among the identified amino acid residues of *Mo*-CBP_2_, 14.5% corresponded to the positively charged arginine (R) and histidine (H), whereas negatively charged, polar uncharged, and non-polar amino acid residues correspond to 1.8, 45.5, and 38.2%, respectively. This is compatible with the cationic property of *Mo*-CBP_2_ (pI = 10.9) and its adsorption to the cation exchange chromatography column equilibrated at pH 5.2, during the purification process.

Searches against the non-redundant protein sequence database of NCBI using BLASTp revealed that *Mo*-CBP_2_ is closely related to *Mo*-CBP_3_ and flocculating proteins from *M. oleifera*, and 2S albumins from different plant sources. Sequence alignment of the six identified *Mo*-CBP_2_ peptides with the sequences of four *Mo*-CBP_3_ isoforms showed 63 to 100% of similarity, except the QPDFQR peptide that aligned exclusively with the isoform 2 (*Mo*-CBP_3_-2), with 83% of similarity. Among the *Mo*-CBP_3_ isoforms, the mean percentage sequence identity was found to *Mo*-CBP_3_-3 (NCBI accession number AHG99684.1), the most abundant of them in the seeds of *M. oleifera* (Freire et al., 2015). With the *M. oleifera* genome recently available (Tian et al., 2015), we tried to localize the nucleotide sequence that corresponds to *Mo*-CBP_2_ using the peptides generated after its tryptic digestion. However, as the primary structures of all *Mo*-CBPs proteins are very similar, it was not possible to predict the *Mo*-CBP_2_ sequence. For instance, it was reported that the flocculent peptides MOCP 2.1 and MOCP 2.2 are distinguished by a single amino acid residue (Shebek et al., 2015). Similar situation might occur with *Mo*-CBP_2_ and other *M. oleifera* proteins. Besides of being a CBP, *Mo*-CBP_3_ is also a member of the 2S albumin family, on the base of their structural similarities (Freire et al., 2015). Therefore, it is reasonable to suggest that *Mo*-CBP_2_ is also a member of the 2S albumin family. The similarities between the *Mo*-CBP_2_ peptide sequences and 2S albumins from *C. masaikai* (63–83%), *B. excelsa* (73%), and *Z. jujuba* (63%) reinforce this hypothesis, although these plant species belong to other botany families different from Moringaceae. 2S albumins are reserve proteins found in mono- or dicotyledonous plants (Youle and Huang, 1981), which apparently are also related to plant defense and some have antifungal activity (Agizzio et al., 2006; Moreno and Clemente, 2008; Cândido et al., 2011), including against *Candida* species (Ribeiro et al., 2012).

The antimicrobial properties of *M. oleifera* have been previously reported. For instance, the aqueous extracts from seeds and other plant parts exhibited antimicrobial activity (Saadabi and Zaid, 2011; Onsare et al., 2013), particularly for Gram positive and Gram negative bacteria rather than for *Candida* spp. Recently, flavonoids extracted from *M. oleifera* seed coat were successfully tested as they exhibited antibiofilm potential against *Staphylococcus aureus* (Gram positive), *Pseudomonas aeruginosa* (Gram negative) and the yeast *C. albicans* (Onsare and Arora, 2015). Moreover, organic extracts from several *M. oleifera* parts also showed antibacterial activity (Brilhante et al., 2015).

Although the antibacterial and anti-*Candida* activities of *M. oleifera* are well known, to the best of our knowledge, this is the first report of a protein from *M. oleifera* seeds with anticandidal activity. Interestingly, all chitin binding proteins (*Mo*-CBP_2_, *Mo*-CBP_3_, and *Mo*-CBP_4_) evaluated in this work exhibited inhibitory activity through *Candida* spp. Comparative analyses between anticandidal effects displayed by *Mo*-CBPs evidenced *Mo*-CBP_2_ as the most potent protein amongst them. *Mo*-CBP_2_ inhibited *C. albicans*, *C. parapsilosis*, *C. krusei*, and *C. tropicalis* growth with MIC_50_ much lower (9.45–37.90 μM) than the other proteins (261.67–310.06 μM). However, much higher concentrations of *Mo*-CBPs (155.84–600.23 μM) were needed to inhibit 90% of fungal growth. *C. krusei* cells were the most sensitive to *Mo*-CBP_2_ (MIC_50_ 9.45 μM and MIC_90_ 155.84 μM). This is an important finding since *C. krusei* is a potentially multi-drug resistant pathogenic yeast due to its intrinsic resistance to fluconazole and tendency to develop reduced echinocandin susceptibility during prolonged therapy and under selection pressure (Scorzoni et al., 2013; Tavernier et al., 2015). The antifungal mode of action of *Mo*-CBP_2_ is probably linked to its ability to disrupt the cell membrane integrity of *C. albicans*, as propidium iodide, which is membrane impermeable, was taken up by cells exposed to *Mo*-CBP_2_, and interacted with nucleic acids as revealed by the appearance of red fluorescence (Wang et al., 2015). Increasing permeability of cell membrane can result from depolarization, disruption of lipid domain organization, pore formation, and unbalance of intracellular electrochemical gradients, leading to loss of membrane functions and cell death (Lee and Lee, 2015; Lee et al., 2015). We hypothesize that the increased permeability of *C. albicans* cell membrane by *Mo*-CBP_2_ exposure might be due to interaction of this protein with chitin, which is one of the major components of fungal cell walls. As suggested for some antifungal peptides, chitin binding ability could help *Mo*-CBP_2_ targets fungal cells efficiently and kill them by disruption of the plasma membrane integrity that increases permeabilization, or by forming pores directly (Bahar and Ren, 2013). *Mo*-CBP_2_ could also gain access to the cell membrane by crossing the cell wall during the exponential growth phase of the yeast cells, when they exhibit increased porosity allowing the passage of compounds up to 70,000 Da (Klis et al., 2014). Once there, *Mo*-CBP_2_, as a basic protein rich in positively charged amino acid residues, could establish electrostatic interaction with the negatively charged cell membrane leading to its disarrangement and cell lysis. Alternatively, *Mo*-CBP_2_ could form transient pores through which it could gain access to the cell interior and enters into contact with intracellular targets (Li et al., 2012; Taniguchi et al., 2013; Choi and Lee, 2014). Regardless whether *Mo*-CBP_2_ gained access or not to the *Candida* cell interior it promoted ROS generation, which are toxic to microorganisms (Mello et al., 2011; Wang et al., 2015). Like for other various antifungal proteins the mechanism of ROS generation after *Mo*-CBP_2_ treatment of *Candida* cells is yet unknown. Nevertheless, ROS are natural compounds produced during the cell metabolism and play important roles in cell signaling and homeostasis, and high levels of ROS generated under environmental stress can result in significant damage to cell structures (Wang et al., 2015). Additionally, excessive ROS damages proteins, lipids, and DNA (Deavall et al., 2012) that besides increasing cell membrane permeability can lead ultimately to cell death. For instance, nystatin, an antifungal, disrupted the cell membrane integrity and induced increased ROS levels in *C. albicans* cells.

Moreover, in the case of internalization into the cell interior, *Mo*-CBP_2_ could interact directly with DNA and exert DNase activity as it broken down *in vitro* the *E. coli* plasmid pUC18, like the commercial recombinant DNase. This finding corroborates with previous studies on the DNase activity of other CBP (Guevara-Morato et al., 2010; Menezes et al., 2014) and 2S albumins from different plant sources (Odintsova et al., 2010; Tomar et al., 2014a,b). Thus, besides its low molecular mass, positive net charge, ability to disrupt cell membrane, and DNase activity, *Mo*-CBP_2_ may also exhibit anticandidal effect by interacting with the genetic material of *C. albicans* cells, leading to its degradation.

In addition to broad spectrum of action on pathogenic microorganisms, low toxicity is a desirable feature of new candidates as antifungal molecules. Hemolytic effect is often considered when antimicrobial safety of new compounds is tested as drugs for human and animal use (Kannan et al., 2013; Sellami et al., 2013; Christoffersen et al., 2015). Although *Mo*-CBP_2_ is toxic to *Candida* spp., this protein did not cause hemolysis in human and rabbit erythrocytes, even in the highest concentration tested (1000 μg/mL). Despite the nystatin effectiveness as antifungal agent and prescription in superficial candidiasis treatment, its clinical use is limited due to its toxicity to human erythrocytes (Bassi and Kaur, 2015). Melittin, other potent antimicrobial peptide against *Candida* spp., causes hemolysis in human blood cells even in low concentrations, which limits its use in antifungal therapy (Lee and Lee, 2015; Lee et al., 2015). Thus, discover of potent antimicrobial agents with high selectivity and reduced or no toxicity to mammalian cells is still a challenge for the scientific community.

In summary, the data presented in this study highlight the potential use of *Mo*-CBP_2_ as an anticandidal agent, based on its ability to inhibit *Candida* spp. growth with apparently low toxicity on mammalian cells.

## Author Contributions

Conceived and designed the study and experiments: JN, MP, JO, LR-B, BR, TG, AM-M, RB, and IV. Performed the experiments: JN, MP, LR-B, TL, HC, JF, ML, and IV. Analyzed the data: JN, MP, JO, LR-B, HC, DS, BR, TG, ML, IV. Contributed reagents/materials/analysis tools: JO, DS, BR, TG, AM-M, RB, and IV. Wrote the paper: JN, MP, JO, LR-B, and IV. All authors reviewed the manuscript.

## Conflict of Interest Statement

The authors declare that the research was conducted in the absence of any commercial or financial relationships that could be construed as a potential conflict of interest.
