# Supplementary material for: A Chitin-binding Protein Purified from Moringa oleifera Seeds Presents Anticandidal Activity by Increasing Cell Membrane Permeability and Reactive Oxygen Species Production
Source: Front Microbiol. 2017 Jun 6;8:980. doi: 10.3389/fmicb.2017.00980 (PMC5459921; doi:10.3389/fmicb.2017.00980)
Supplement: Supplementary file 1 [file Image_1.PDF]

Supplementary Material

A chitin-binding protein purified from *Moringa oleifera* seeds presents anticandidal activity by increasing cell membrane permeability and reactive oxygen species production

João X. Silva Neto[1], Mirella L. Pereira[1], Jose T. A. Oliveira[1], Lady C. B. Rocha-Bezerra[1], Tiago D. P. Lopes[1], Helen P. S. Costa[1], Daniele O. B. Sousa[1], Bruno A. M. Rocha[1], Thalles B. Grangeiro[2], José E. C. Freire[1], Ana Cristina O. Monteiro-Moreira[3], Marina D. P. Lobo[1,3], Raimunda S. N. Brilhante[4] and Ilka M. Vasconcelos[1*]

*** Correspondence:** Corresponding Author: imvasco@ufc.br

1 Supplementary Figures

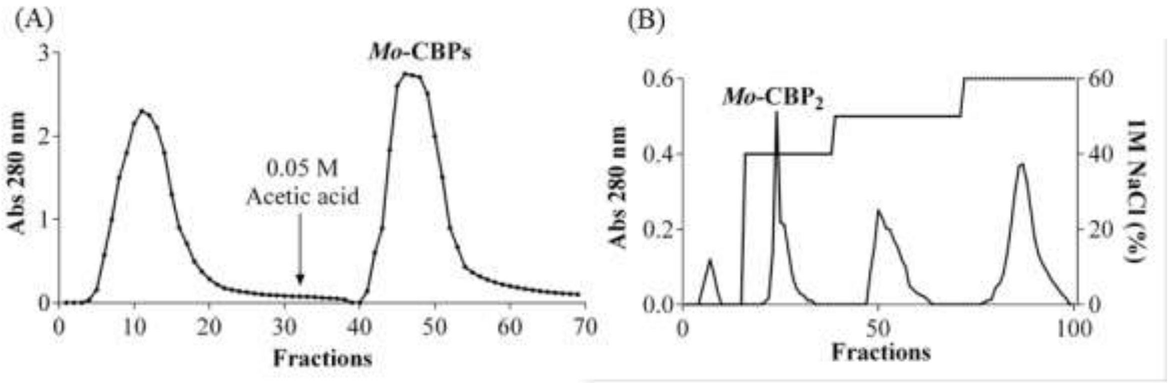

Supplementary Figure 1. Chromatographic steps of *Mo*-CBP$_2$ purification. **(A)** Affinity chromatography: the seed albumin fraction (1.0 g) was applied on a chitin column equilibrated with 0.05 M Tris-HCl buffer, pH 8.0, containing 0.15 M NaCl. The non-adsorbed proteins were eluted with the equilibrium buffer. The chitin-binding proteins (*Mo*-CBPs) were eluted with 0.05 M acetic acid. Protein fractions (2.0 mL) were collected at 60 mL/h flow rate. **(B)** Cation exchange chromatography: *Mo*-CBPs (400 mg) were loaded on a CM-Sepharose™ Fast Flow column equilibrated with 0.05 M sodium acetate buffer, pH 5.2. *Mo*-CBP$_2$ was eluted with equilibration buffer containing 0.4 M NaCl. Fractions (4.5 mL) were collected at 45 mL/h flow rate. In both chromatographic steps, eluted proteins were monitored at 280 nm.

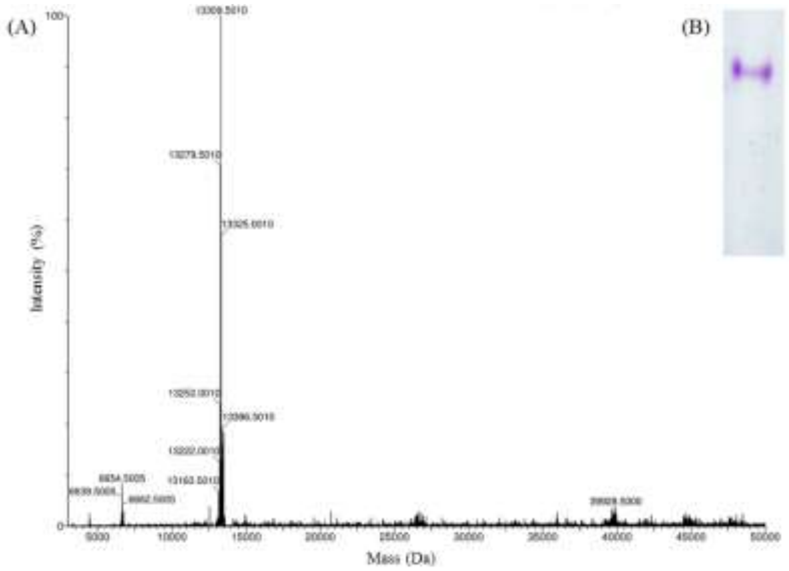

Supplementary Figure 2. *ESI-mass spectrum* of *Mo*-CBP_2 under native conditions. Inset: Native-PAGE (15%) of *Mo*-CBP_2 (5.0 µg).

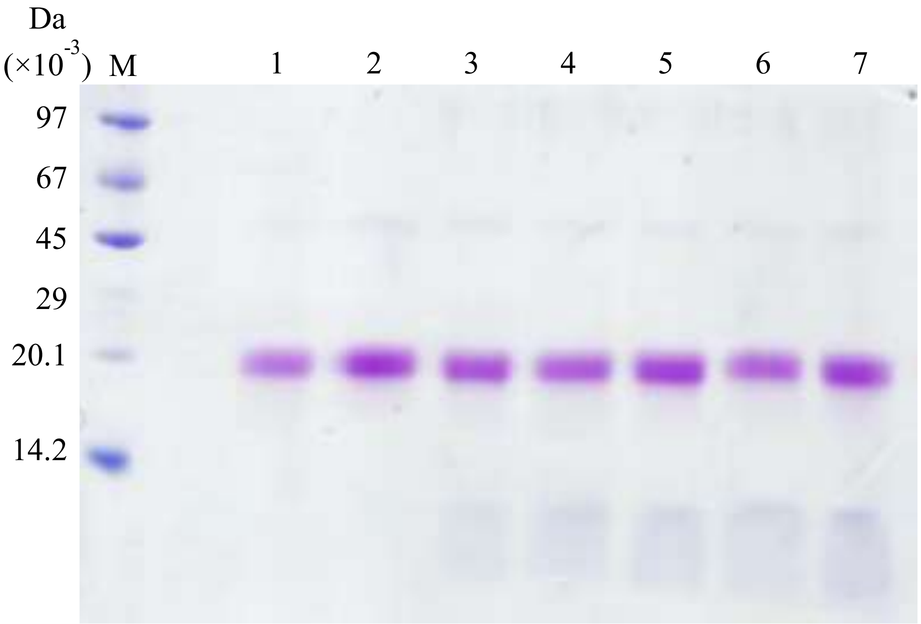

Supplementary Figure 3. SDS-PAGE (15%). Lane M = protein molecular mass markers; lane 1= *Mo*-CBP_2 (5.0 µg); lanes 2-4 = *Mo*-CBP_2 (5.0 µg) treated with 4, 6, and 8% (v/v) 2-ME, respectively; lanes 5-7 = *Mo*-CBP_2 (5.0 µg) treated with 0.001, 0.01, and 0.1 M DTT followed by 0.002, 0.02, and 0.2 M IAA, respectively.

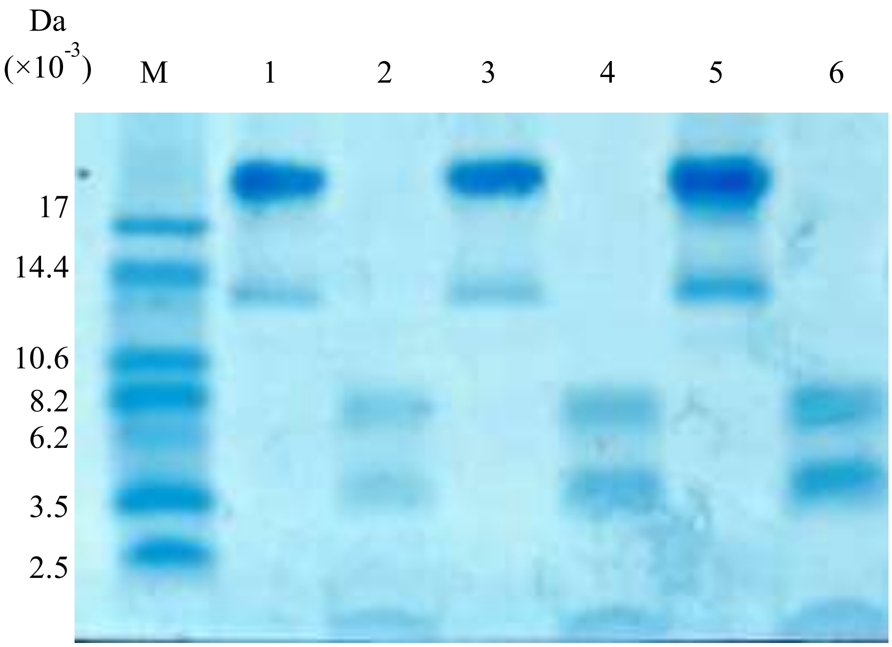

Supplementary Figure 4. Tricine-SDS-PAGE. Lane M = protein molecular mass markers; lanes 1, 3, and 5 = *Mo*-CBP$_2$ (10.0 µg); lanes 2, 4, and 6 = *Mo*-CBP$_2$ (10.0 µg) treated with 0.1 M DTT and 0.2 M IAA followed by 4% (v/v) 2-ME.

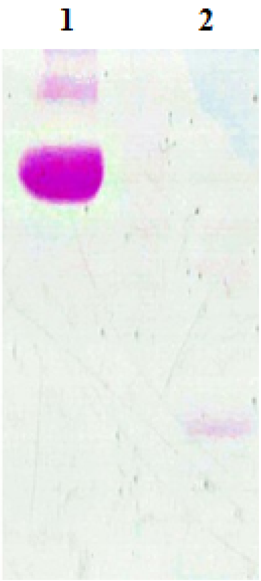

Supplementary Figure 5. Glycoprotein nature of *Mo*-CBP$_2$ assessed by SDS-PAGE (15%). Lane 1 = fetuin (30.0 µg), lane 2 = *Mo*-CBP$_2$ (15.0 µg). Both proteins were stained with the periodic acid-Schiff reagent.
